# Swimming attenuates inflammation, oxidative stress, and apoptosis in a rat model of dextran sulfate sodium-induced chronic colitis

**DOI:** 10.18632/oncotarget.14080

**Published:** 2016-12-21

**Authors:** Ling Qin, Zhi-qiang Yao, Qi Chang, Ya-li Zhao, Ning-ning Liu, Xiao-shan Zhu, Qin-qin Liu, Li-feng Wang, An-gang Yang, Chun-fang Gao, Jun-tang Li

**Affiliations:** ^1^ Department of Hematology, First Affiliated Hospital, and College of Clinical Medicine of Henan University of Science and Technology, Luoyang, Henan, China; ^2^ Centre of Inflammation and Cancer Research, 150th Central Hospital of PLA, Luoyang, Henan, China; ^3^ Centre of Biomaterial and Biophysics Research, Institute of Training Medicine, 150th Central Hospital of PLA, Luoyang, Henan, China; ^4^ State Key Laboratory of Cancer Biology, Department of Biochemistry and Molecular Biology, Fourth Military Medical University, Xian, Shaanxi, China; ^5^ State Key Laboratory of Cancer Biology, Department of Immunology, Fourth Military Medical University, Xian, Shaanxi, China

**Keywords:** chronic colitis, swimming, inflammation, oxidative stress, apoptosis, Immunology and Microbiology Section, Immune response, Immunity

## Abstract

Increasing evidence suggests that regular physical exercise suppresses chronic inflammation. However, the potential inhibitory effects of swimming on dextran sulfate sodium (DSS)-induced chronic colitis, and its underlying mechanisms, remain unclear. In this study, rats were orally administered DSS to induce chronic colitis, and subsequently treated with or without swimming exercise. A 7-week swimming program (1 or 1.5 hours per day, 5 days per week) ameliorated DSS-caused colon shortening, colon barrier disruption, spleen enlargement, serum LDH release, and reduction of body weight gain. Swimming for 1.5 hours per day afforded greater protection than 1 hour per day. Swimming ameliorated DSS-induced decrease in crypt depth, and increases in myeloperoxidase activity, infiltration of Ly6G^+^ neutrophils and TNF-a- and IFN-?-expressing CD3^+^ T cells, as well as fecal calprotectin and lactoferrin. Swimming inhibited pro-inflammatory cytokine and chemokine production and decreased the protein expression of phosphorylated nuclear factor-?B p65 and cyclooxygenase 2, whereas it elevated interleukin-10 levels. Swimming impeded the generation of reactive oxygen species, malondialdehyde, and nitric oxide; however, it boosted glutathione levels, total antioxidant capacity, and superoxide dismutase and glutathione peroxidase activities. Additionally, swimming decreased caspase-3 activity and expression of apoptosis-inducing factor, cytochrome c, Bax, and cleaved-caspase-3, but increased Bcl-2 levels. Overall, these results suggest that swimming exerts beneficial effects on DSS-induced chronic colitis by modulating inflammation, oxidative stress, and apoptosis.

## INTRODUCTION

Chronic inflammation plays a major role in the pathology of various diseases. Inflammatory bowel disease (IBD), including Crohn's disease and ulcerative colitis (UC), is characterized by an inappropriate and continuous active inflammatory response accompanied by tissue destruction [1]. Conventional IBD treatments can reduce active disease periods and help to maintain remission, but these treatments often cause side effects, show marginal results, and induce resistance to therapy. Therefore, alternative treatment strategies are urgently needed.

Dextran sulfate sodium (DSS) is a non-genotoxic sulfated polysaccharide that is frequently used to induce experimental chronic colitis, which is histopathologically similar to human UC [2]. Intestinal epithelial barrier disruption is regarded as the main event during progression of IBD; this is followed by activation of the immune response [3]. Activated immune responses in the intestine result in the excessive production of pro-inflammatory cytokines, such as tumor necrosis factor-α (TNF-α), interleukin (IL)-1β, IL-6, IL-8, and interferon-γ (IFN-γ). These cytokines amplify inflammatory cascades by triggering the generation of other pro-inflammatory cytokines and chemokines, thereby recruiting neutrophils to the site of mucosal injury. Activated neutrophils produce fecal biomarkers of IBD such as calprotectin and lactoferrin [4], and generate large amounts of reactive oxygen species (ROS), nitric oxide (NO), and prostaglandin E_2_ (PGE_2_), which ultimately cause mucosal disruption [3]. Oxidative stress plays a prominent role in the pathogenesis of chronic colitis [5]. Excessive ROS activate nuclear factor-κB (NF-κB), which in turn induces the production of numerous inflammatory mediators as well as the expression of inducible nitric oxide synthase (iNOS) and cyclooxygenase 2 (COX2) [6]. The pathogenesis of IBD also involves increased apoptosis with consequent loss of intestinal epithelial cells [7].

Regular physical activity improves overall well-being and may be used as an adjunct anti-inflammatory therapy for chronic inflammatory diseases in both humans and animals [8]. Cook et al. [9] established a mouse colitis model to show that forced treadmill exercise exacerbates inflammation, whereas voluntary wheel training is protective. Previous studies indicate that voluntary wheel exercise inhibits apoptosis of splenic lymphocyte subsets in mice by modulating the immune system and increasing antioxidant capacity [10]. In particular, swimming has been prescribed as a non-pharmacological treatment for arterial hypertension, obesity, and coronary heart disease as it reduces the levels of inflammatory cytokines without causing oxidative stress [11]. Also, swimming reduces the risk of dimethylhydrazine-induced colon cancer in rats by inhibiting inflammation [12]. All of these findings encouraged us to examine the potential protective effects of swimming exercise, and the underlying mechanisms, in rats with DSS-induced colitis, an experimental model of human IBD.

In this study, we found that swimming attenuated DSS-induced colon shortening, decrease of crypt depth, colonic barrier disruption, spleen swelling, LDH release in blood, decrease in body weight (BW) gain, histopathological deteriation, the increases in fecal biomarkers of IBD, and the immigration of Ly6G^+^ neutrophils and TNF-α- and IFN-γ-expressing CD3^+^ T cells into mesenteric lymph nodes (MLNs) and the lamina propria (LP). Examination of the underlying molecular mechanisms revealed that swimming reduced the expression of phosphorylated NF-κB p65 and COX2 in the DSS-treated rat colon, as well as the levels of TNF-α, IL-1β, IL-6, IL-10, KC, CCL2, and PGE_2_. Moreover, swimming decreased the production of ROS, malondialdehyde (MDA), and NO, and inhibited iNOS activity, while increased glutathione (GSH) and total antioxidant capacity (TAC) levels, and the activities of superoxide dismutase (SOD) and glutathione peroxidase (GPx). Swimming also impeded DSS-induced apoptosis in the colon by inhibiting caspase-3 activity, reducing the expression of apoptosis-inducing factor (AIF), cytochrome c, Bax, and cleaved-caspase-3 (Cl-caspase-3), and increasing Bcl-2 level. These results indicate that swimming exerts anti-inflammatory, antioxidant, and anti-apoptotic effects, thereby ameliorating DSS-caused chronic colitis.

## RESULTS

### Swimming attenuates DSS-induced spleen enlargement, reduction of BW gain, and colon injury

**Figure 1:**
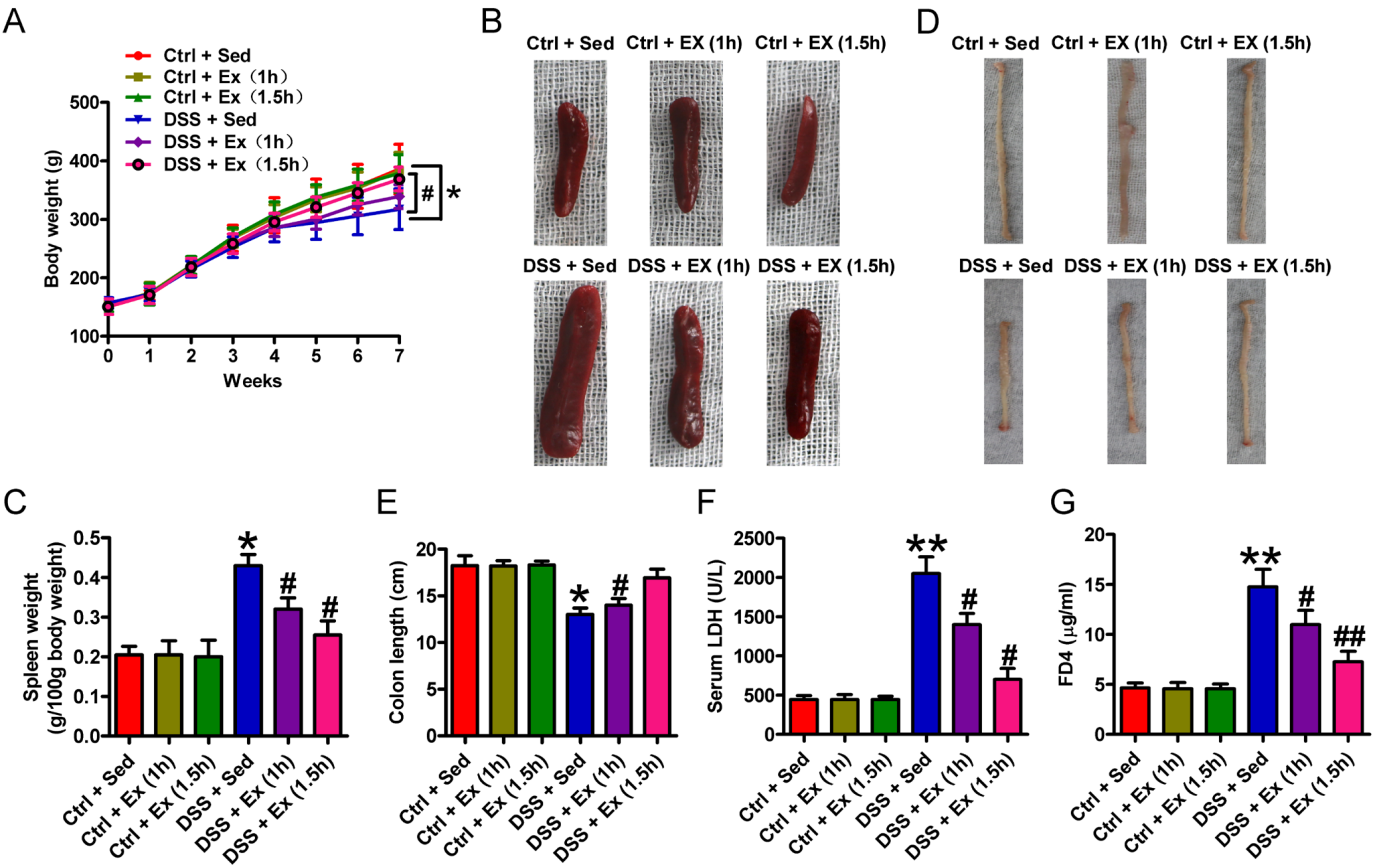
Effects of swimming on the severity of DSS-induced colitis in rats **A**. Changes in body weight. Body weight was measured both before and after adaptation, and once per week throughout the experimental period. **B**.-**F**. After 7 weeks of swimming exercise, rats were fasted for 4 h and then euthanized with sodium pentobarbital. The spleen and entire colon were removed, and blood was collected. Representative photo of spleens **B**., changes in spleen weight **C**., representative macroscopic view of the colons **D**., quantification of colon length **E**., and measurement of serum LDH levels **F**.. **G**. Changes in colon barrier function. After 4 h of fasting, rats were administered FD4 by gavage. Plasma was sampled 5 h after the procedure, and its fluorescence was measured. Data are representative of three independent experiments and are expressed as the mean ± SD. *P < 0.05 and ^*^P < 0.01 *vs*. Ctrl groups; ^#^*P* < 0.05 and ^##^*P* < 0.01 *vs*. DSS + Sed group.

**Figure 2:**
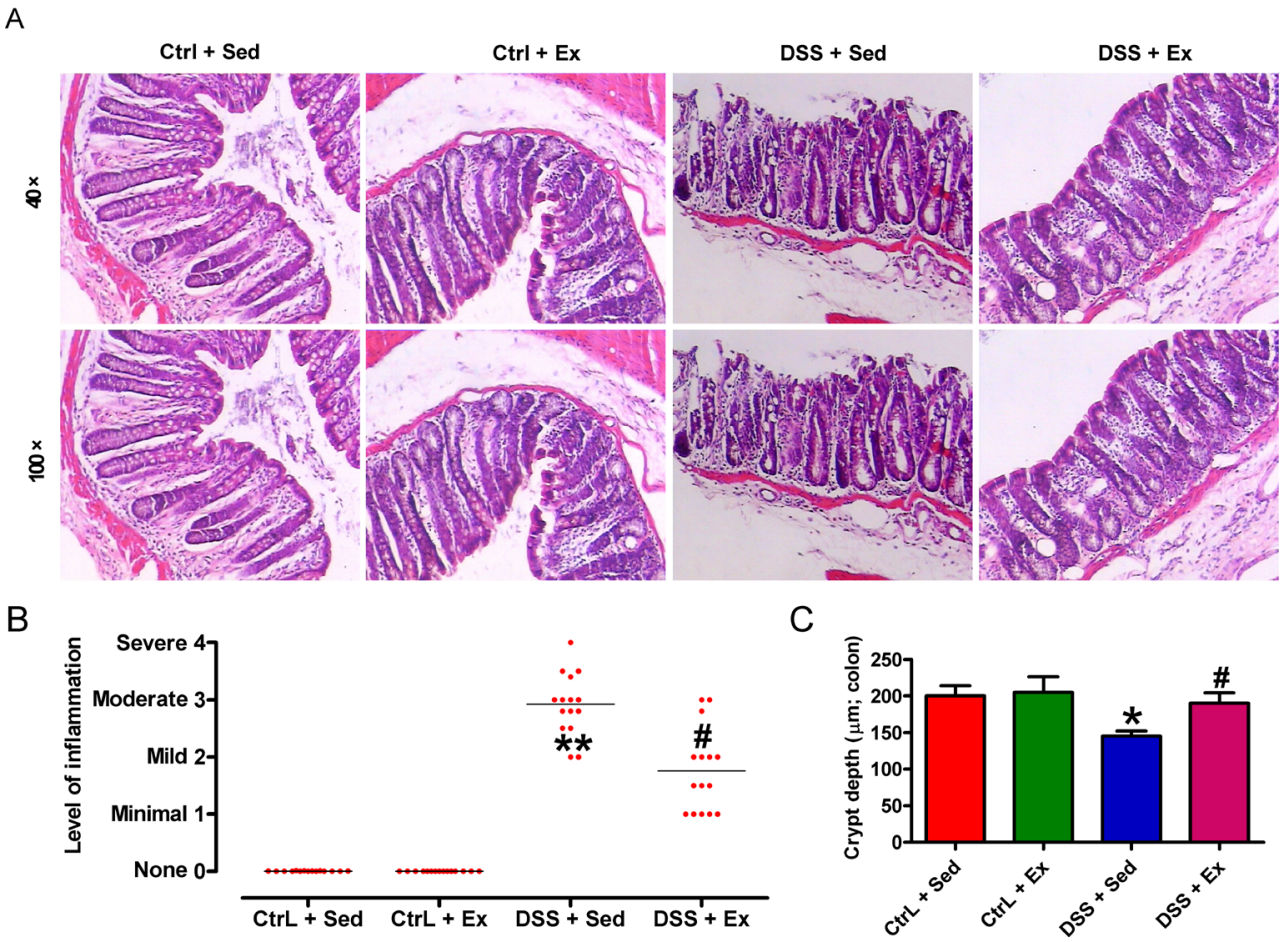
Swimming attenuates DSS-induced histopathological damage in the rat colon After 7 weeks of swimming exercise, colon sections were collected and utilized for histopathological investigation. **A**. Representative images of HE-stained colon tissues (40× magnification, upper panels; 100× magnification, lower panels). **B**. Inflammation scores. Results are presented as a dot plot, with the lines representing the mean inflammation score. Each dot represents an individual rat. **C**. Crypt depth in colon mucosa sections. Data are representative of three independent experiments and are expressed as the mean ± SD. *P < 0.05 and ^*^P < 0.01 *vs*. Ctrl groups; ^#^*P* < 0.05 *vs*.DSS + Sed group.

We initially found that oral administration of DSS caused significant decrease in BW gain (Figure 1A), spleen swelling (Figures 1B and 1C), elevated serum LDH (Figure 1D), colon shortening (Figure 1E), and colonic barrier disruption (Figure 1F) in rats when compared with control (Ctrl) animals. Swimming for 1 hour per day (h/d) or 1.5 h/d led to a significant inhibition of the above vicious effects in DSS-insulted rats (Figures 1A-1G); however, swimming for 1.5 h/d had a greater protective effect. Thus, a period of 1.5 h/d was selected for further studies. As shown in Figures 2A and 2B, the DSS + Sedentary (Sed) group showed severe dysplastic lesions in colon tissues and a marked increase in the inflammation score when compared with the Ctrl groups; however, swimming reduced inflammation-led flat dysplasia and improved the colonic architecture in DSS-challenged rats. Moreover, DSS-treated rats exhibited decreased crypt depth, which was reversed by swimming (Figure 2C). These results indicate that swimming ameliorates the decreased BW gain, spleen enlargement and colon damage in DSS-administrated rats.

### Swimming reduces the infiltration of activated neutrophils and CD3^+^ T cells into colon in DSS-induced colitis

**Figure 3:**
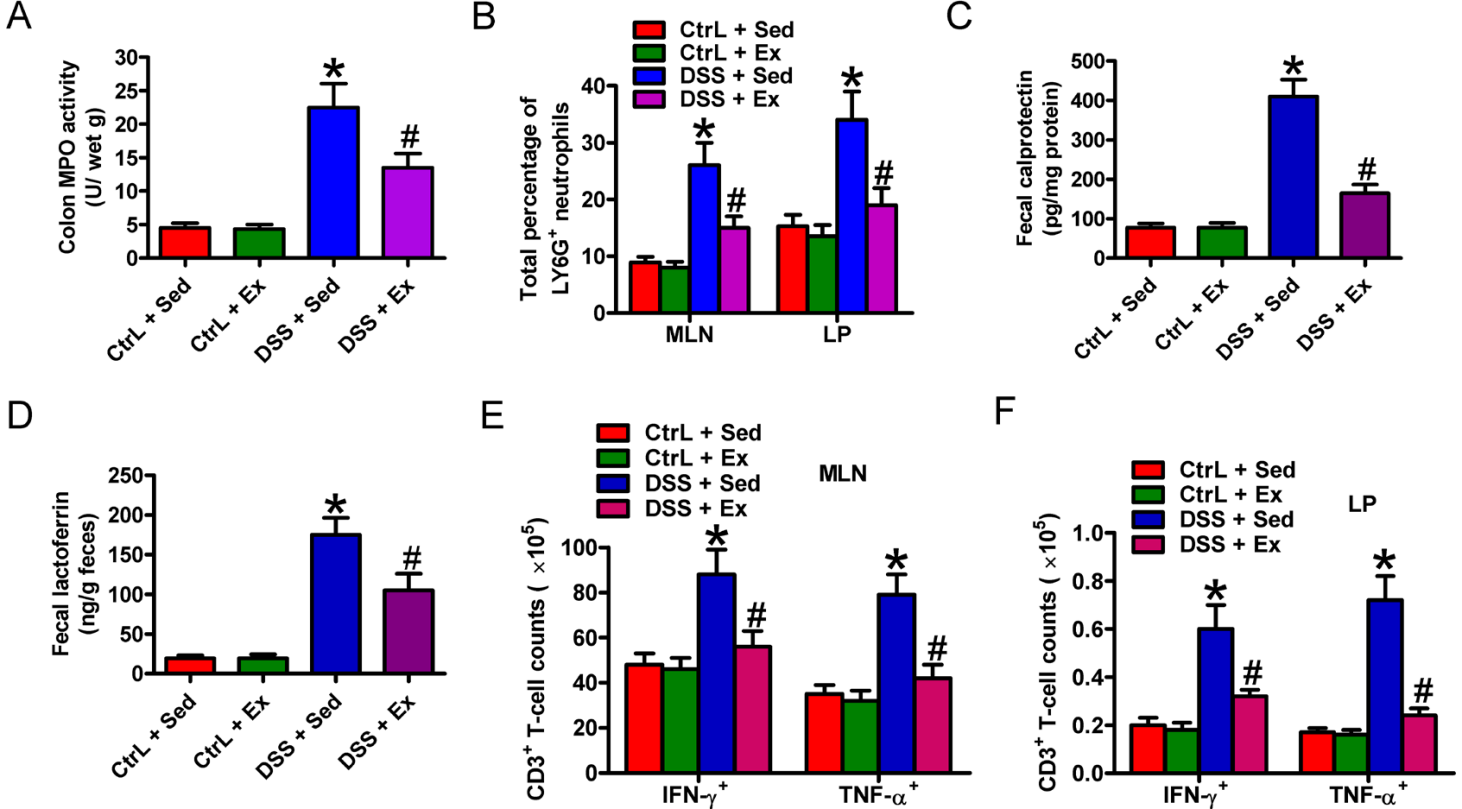
Swimming reduces MPO activity, infiltration of Ly6G **^+^** neutrophil and TNF-α- and IFN-γ-expressing CD3**^+^** T cells and fecal biomarkers in DSS-induced colitis in rats. **A**. MPO activity in colon tissues from the different treatment groups. **B**. Total percentage of Ly6G^+^ neutrophils in the MLNs and LPs. **C**. and **D**. Measurement of fecal biomarkers such as calprotectin **C**. and lactoferrrin **D**.. Feces were harvested and ELISAs were performed to determine the levels of calprotectin and lactoferrrin. **E**. and **F**. Changes in the number of TNF-α- and IFN-γ-expressing CD3^+^ T cells in the MLNs **E**. and LP **F**.. Lymphocytes were freshly isolated from the MLNs and LP and stained to quantify CD3^+^ T cells and neutrophils. MLN- or LP-derived CD3^−^ lymphocytes were examined for Ly6G expression by flow cytometry. Data are representative of three independent experiments and are expressed as the mean ± SD. *P < 0.05 *vs*. Ctrl groups; ^#^*P* < 0.05 *vs*. DSS + Sed group.

Neutrophils play an important role in DSS-induced colonic mucosal injury [13]. As shown in Figures 3A and 3B, DSS caused a marked enhancement in myeloperoxidase (MPO) activity in colon tissues, and in the percentage of Ly6G^+^ neutrophils in the MLNs and LPs, both of which were significantly attenuated by swimming. Fecal calprotectin and lactoferrin, considered as biomarkers of IBD, increases significantly as infiltration of neutrophils in intestinal tracts [14]. Here, DSS administration increased fecal calprotectin and lactoferrin compared to Ctrl groups; however, swimming effectively hindered the elevation (Figures 3C and 3D). Next, we investigated the effect of swimming on the recruitment of activated CD3^+^ T cells into MLNs and LPs after DSS challenge. Flow cytometry analysis revealed that the numbers of TNF-α- and IFN-γ-expressing CD3^+^ T cells increased after DSS treatment; these increases were significantly reduced by swimming (Figures 3E and 3F). These results suggest that swimming inhibits neutrophil and CD3^+^ T cell infiltration into colon in DSS-induced colitis.

### Swimming modulates the production of inflammatory mediators in DSS-induced colitis

**Figure 4:**
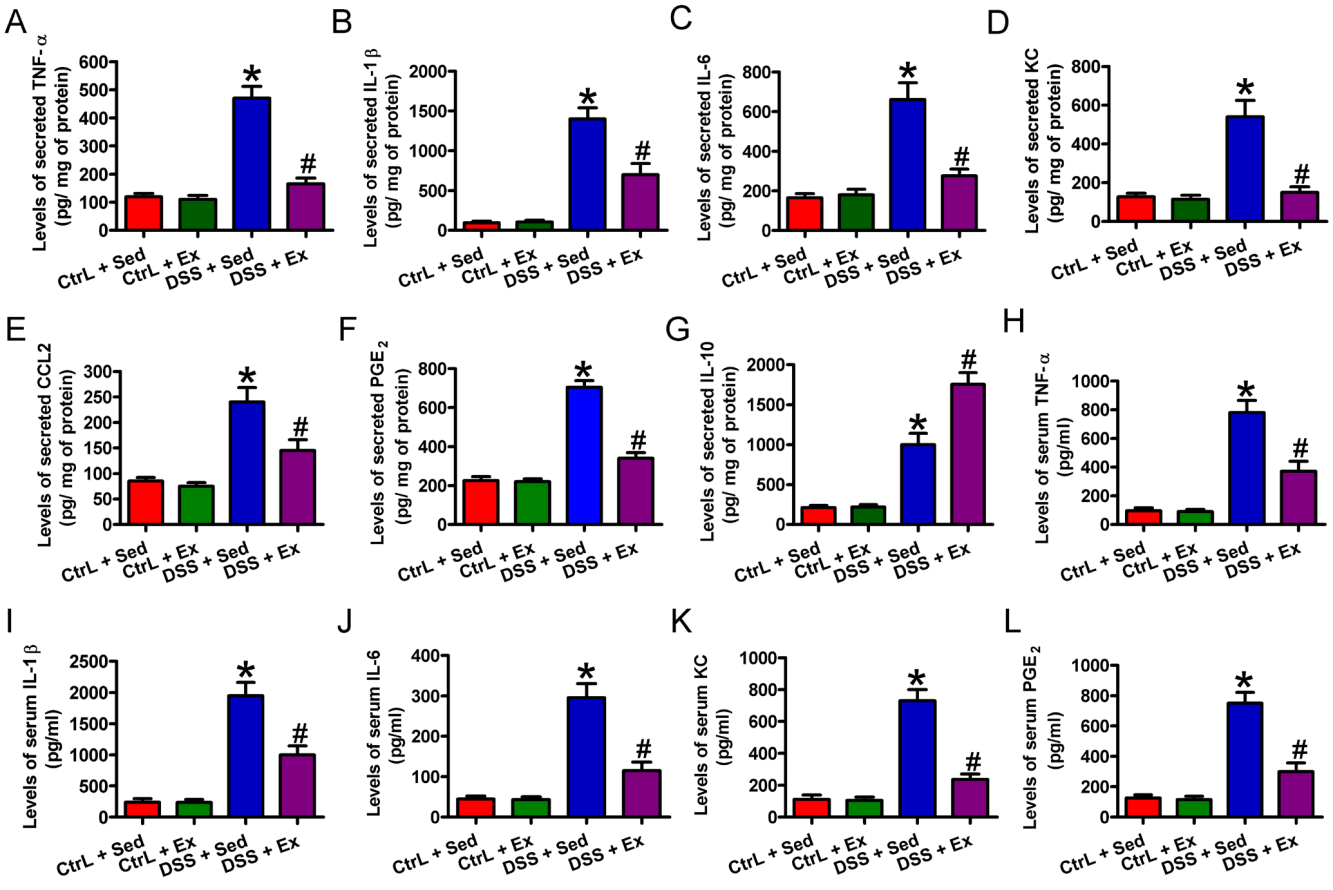
Swimming inhibits the production of inflammatory mediators in rats with DSS-induced colitis After 7 weeks of swimming exercise, the colon was removed from the distal end of the cecum to the rectum, longitudinally cut, thoroughly rinsed in sterile 20 mM PBS (pH 7.4), spread onto the bottoms of six-well dishes, and incubated overnight in 1 mL of culture medium. **A**.-**G**. ELISAs were performed to detect the supernatant levels of TNF-α **A**., IL-1β **B**., IL-6 **C**., KC **D**., CCL2 **E**., PGE_2_ **F**., and IL-10 **G**.. After 7 weeks of swimming exercise, serum was collected from every group. **H**.-**L**. ELISAs were carried out to detect the serum levels of TNF-α **H**., IL-1β **I**., IL-6 **J**., KC **K**., PGE_2_ **L**.. Data are representative of three independent experiments and are expressed as the mean ± SD. *P < 0.05 *vs*. Ctrl groups; ^#^*P* < 0.05 *vs*. DSS + Sed group.

To gain insight into the inflammatory milieu associated with DSS-induced colitis, we performed enzyme-linked immunosorbent assays (ELISAs) to measure the levels of various inflammatory mediators. DSS induced severe inflammatory responses, as indicated by markedly higher levels of TNF-α (Figure 4A), IL-1β (Figure 4B), IL-6 (Figure 4C), KC (Figure 4D), CCL2 (Figure 4E), and PGE_2_ (Figure 4F) in the colon of rats than in that of Ctrl rats; these increases were significantly attenuated by swimming. DSS treatment increased IL-10 production, however, which was further enhanced by swimming (Figure 4G). Moreover, DSS challenge increased rat serum levels of TNF-α (Figure 4H), IL-1β (Figure 4I), IL-6 (Figure 4J), KC (Figure 4K), and PGE_2_ (Figure 4L), which were significantly inhibited by swimming. These results demonstrate that swimming exerts anti-inflammatory effects by inhibiting the production of several pro-inflammatory mediators and increasing IL-10 levels.

### Swimming abrogates expression of NF-κB p65 and COX2

**Figure 5:**
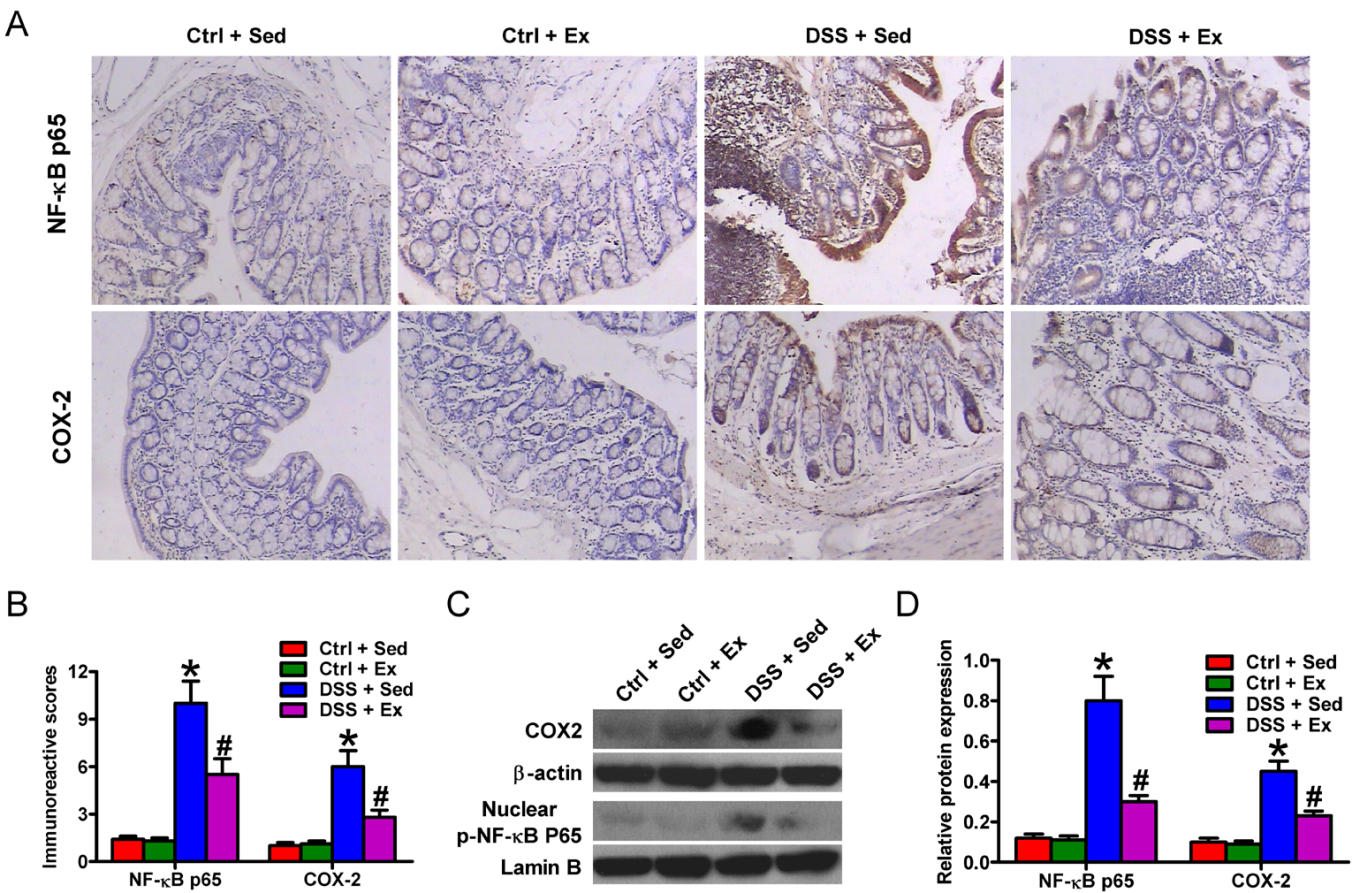
Swimming decreases the expression of NF-κB p65 and COX2 protein in the colon of rats with DSS-induced colitis After 7 weeks of swimming exercise, colon tissues were collected and used for immunohistochemical investigation, or were homogenized in tissue lysis buffer prior to Western blot analysis. **A**. Representative images of IHC-stained tissues showing NF-κB p65 and COX2 expression (100× magnification). **B**. Immunoreactive scores of the NF-κB p65 and COX2 shown in **A**.. **C**. Representative blots showing COX2 and p-NF-κB p65 expressions after Western blot analysis. β-actin and lamin B were used as endogenous loading controls. **D**. The COX2/β-actin and p-NF-κB p65/lamin B ratios. Data are representative data of three independent experiments and are expressed as the mean ± SD. *P < 0.05 *vs*. Ctrl groups; ^#^*P* < 0.05 *vs*. DSS + Sed group.

NF-κB and COX2 play crucial roles in the pathogenesis of IBD [6]. Immunohistochemistry (IHC) staining of colon tissue from rats with DSS-induced colitis revealed extensive NF-κB p65 and COX2 expression (Figures 5A and 5B). Western blot analysis confirmed that DSS induced a significant increase in the expression of phosphorylated (p)-NF-κB p65 and COX2 (Figures 5C and 5D). Notably, swimming led to a significant reduction in the levels of NF-κB p65 and COX2 (Figures 5A-5D). These results indicate that downregulation of NF-κB p65 and COX2 may underlie the beneficial effects of swimming in rats with DSS-induced colitis.

### Swimming inhibits oxidative stress and increases antioxidant products

**Figure 6:**
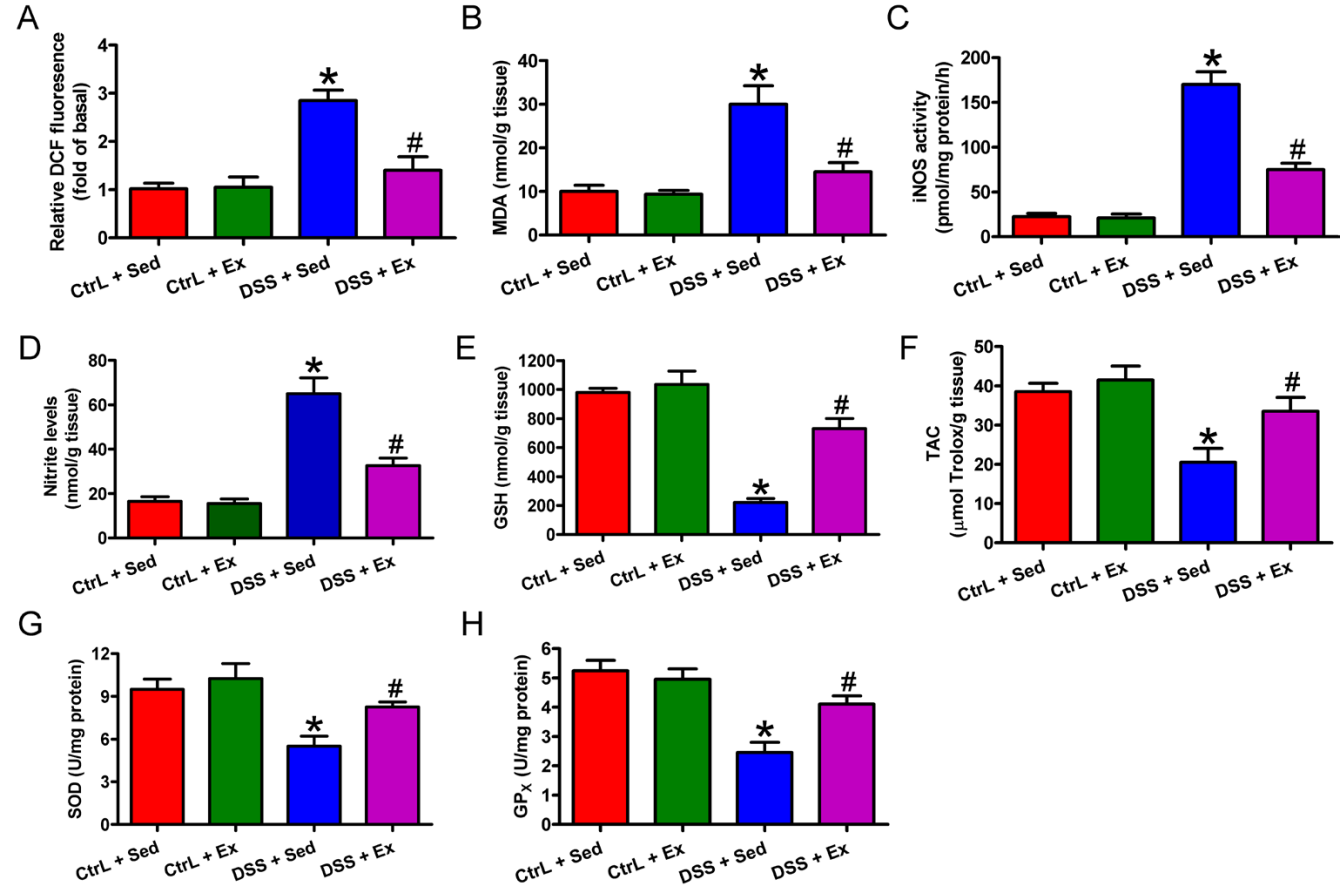
Swimming ameliorates oxidative stress and increases antioxidant defense in the colon of rats with DSS-induced colitis After 7 weeks of swimming exercise, colon sections were collected and homogenized in tissue lysis buffer and centrifuged, and the supernatants were harvested. **A**. ROS production was measured in terms of relative DCF fluorescence. **B**. Lipid peroxide was measured in terms of MDA production. **C**. iNOS activity was measured in a standard assay that monitors the conversion of arginine to citrulline. **D**. Nitrite production was assessed in a colorimetric reaction with the Griess reagent. **E**. GSH levels were determined using DTNB reagent. **F**. TAC was determined using a commercial antioxidant assay kit. **G**. SOD activity was assessed according to inhibition of pyrogallol auto-oxidation. **H**. GPx activity was determined according to its ability to oxidize GSH. Data are representative of three independent experiments and are expressed as the mean ± SD. *P < 0.05 *vs*. Ctrl groups; ^#^*P* < 0.05 *vs*. DSS + Sed group.

During the development of IBD, the inflammatory process induces oxidative stress and reduces cellular antioxidant capacity [6]. DSS caused marked oxidative stress, as indicated by increases in ROS (Figure 6A), MDA (Figure 6B), and NO (Figure 6C) levels, and iNOS activity (Figure 6D). This was accompanied by decreases in GSH (Figure 6E) and TAC (Figure 6F) contents, and SOD (Figure 6G) and GPx (Figure 6H) activities. Swimming provided a significant protection against oxidative stress, as evidenced by the decreased levels of ROS, MDA, and NO, and reduced iNOS activity (Figures 6A-6D), in addition to the rescuing of GSH and TAC levels and of SOD and GPx activities (Figures 6E-6H). Thus, swimming attenuates oxidative stress and boosts antioxidant defenses to protect against DSS-induced colitis.

### Swimming inhibits apoptosis in rats with DSS-induced colitis

**Figure 7:**
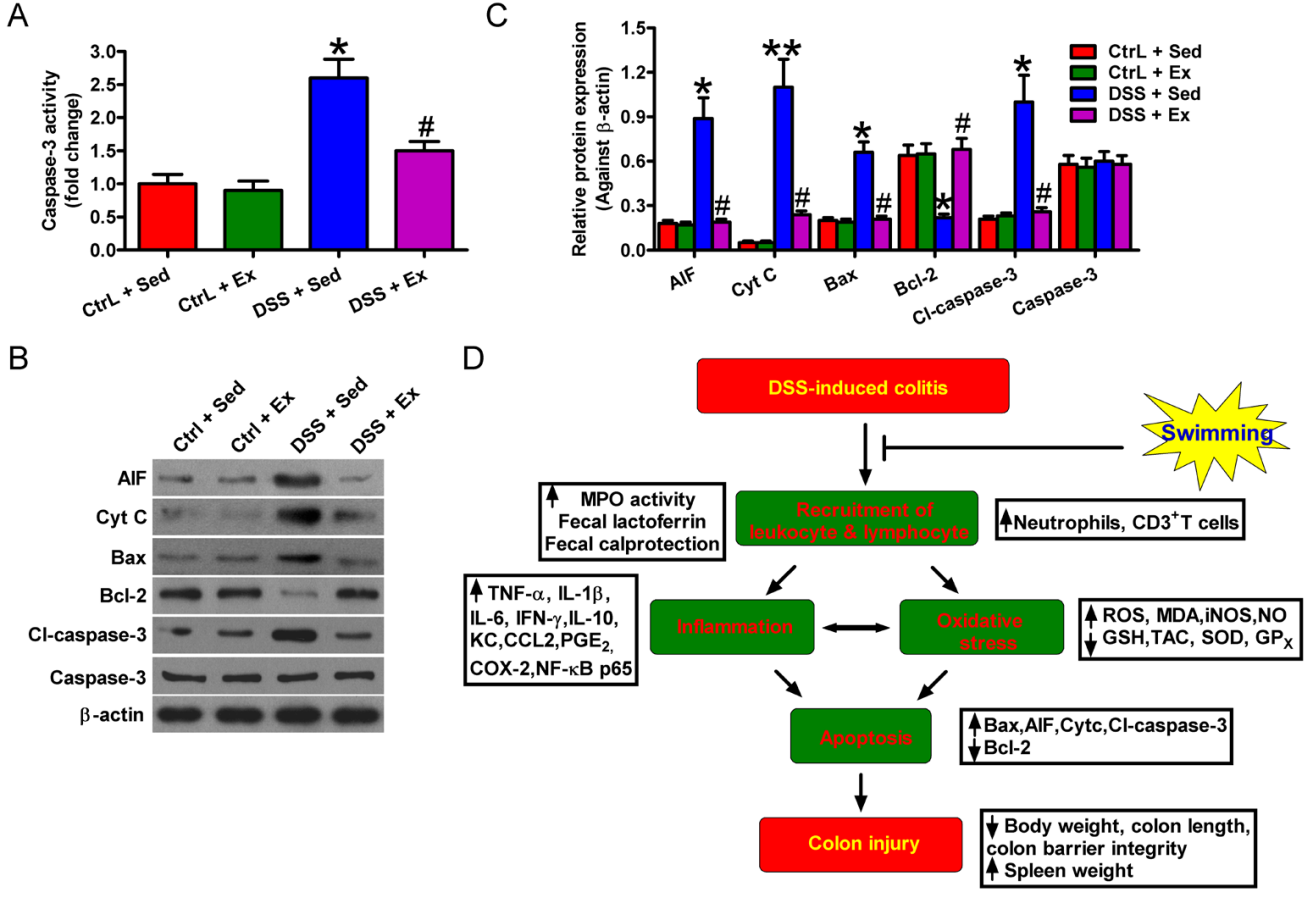
Swimming impedes apoptosis in the colon of rats with DSS-induced colitis After 7 weeks of swimming exercise, colon sections were collected, homogenized in tissue lysis buffer, and centrifuged, and the supernatants were harvested. **A**. Caspase-3 activity was measured using a Caspase-3/CPP32 colorimetry kit. **B**. Expression of AIF, cytochrome c, Bax, Bcl-2, Cl-caspase-3, and caspase-3 proteins was assessed by Western blotting. β-actin was used as an endogenous loading control. **C**. The relative expression of the proteins in **B**. was normalized against β-actin. **D**. Diagram depicting the inhibitory effects of swimming exercise on DSS-induced colitis in rats. Data are representative data of three independent experiments and are expressed as the mean ± SD. *P < 0.05 and ^*^P < 0.01 *vs*. Ctrl groups; ^#^*P* < 0.05 *vs*. DSS + Sed group.

Exposure of the colonic mucosa to intracellular stressors such as ROS in an inflammatory environment triggers apoptosis of colon epithelial cells, leading to progression of IBD [15]. Thus, we investigated the effects of swimming on apoptosis of colon epithelial cell in rats with DSS-induced colitis. We found that DSS induced apoptosis of inflamed colon epithelial cells, as indicated by increases in caspase-3 activity (Figure 7A) and expression of Cl-caspase-3, AIF, cytochrome c, and Bax, along with downregulation of Bcl-2 (Figures 7B and 7C). Swimming counteracted the above changes, thereby promoting cell survival (Figures 7A-7C). These findings suggest that swimming protects the colon mucosa from apoptosis in DSS-induced colitis.

## DISCUSSION

In this study, we demonstrate that swimming alleviates DSS-induced colitis in rats. The beneficial effects are as follows (Figure 7D): (1) Attenuation of DSS-induced reduced BW gain, spleen swelling, serum LDH release, colon shortening, decreased crypt depth, and colonic barrier disruption; (2) reduction of neutrophil and TNF-α- and IFN-γ-expressing CD3^+^ T cell infiltration into MLNs and LPs and fecal calprotectin and lactoferrin; (3) inhibition of inflammatory mediator production and NF-κB p65 and COX2 protein expression; (4) decrease in oxidative stress and increase in antioxidant defense; and (5) downregulation of pro-apoptotic molecules and upregulation of Bcl-2 expression.

IBD is associated with an influx of neutrophils into the mucosa and, subsequently, into the intestinal lumen, resulting in crypt abscess formation [16]. Monoclonal antibody-mediated depletion of neutrophils reduces several parameters associated with DSS-induced colitis in rats [13]. Morohoshi et al. [17] demonstrated significant increases in neutrophil elastase activity in both plasma and colonic mucosal tissues in UC patients, and showed that ONO-5046 (a specific inhibitor of neutrophil elastase) has therapeutic effects against DSS-induced colitis by reversing weight loss and spleen enlargement and reducing inflammation scores. Here, we found that MPO activity, an indicator of neutrophil infiltration, was significantly higher in rats with DSS-induced colitis than in controls; these increases were attenuated by swimming. These observations were confirmed by experiments demonstrating a reduction in the percentage of Ly6G^+^ neutrophils in the MLNs and LPs. Fecal biomarkers of IBD such as calprotectin and lactoferrin increase significantly as infiltration of neutrophils in intestinal tracts [14]. Consistent with the previous studies, we showed that DSS induced increases in calprotectin and lactoferrin in rat feces, which were reduced by swimming. Thus, mitigation of neutrophil influx may be one reason that swimming protects against colon injury.

Consistent with the known anti-inflammatory and antioxidant effects of regular physical exercise, we found that regular swimming significantly reduced colon levels of TNF-α, IL-1β, IL-6, KC, NO, PGE_2_, and COX2 and serum levels of TNF-α, IL-1β, IL-6, KC, and PGE_2_ in DSS-treated rats, which was accompanied by increased IL-10 production. TNF-α, IL-1β, IL-6, NO, and PGE_2_ are key mediators of IBD [18, 19]. Increased levels of IL-10 are observed in the plasma of IBD patients and in the colon of rats with DSS-induced colitis; IL-10 attenuates the exaggerated inflammatory response [20, 21]. It was reported that exercise increased IL-10 levels in DSS-induced colitis in adiponectin deficient mice and in TNBS-induced colitis in rats [22, 23]. IL-10 elevation is thought to arise *via* a compensatory mechanism because this cytokine reduces mucosal inflammation [24, 25]. The detailed mechanism(s) by which swimming modulates the expression of these molecules requires further investigation. Recent studies indicate that regular exercise inhibits the activation of NF-κB, which transcriptionally regulates COX2 and iNOS [26]. COX2 generates PGE_2_, which provokes intestinal hyperemia and edema. iNOS activation releases a surplus of NO, which undermines colon integrity *via* the synthesis of peroxynitrite, a potent oxidizing agent formed during the reaction of NO with the superoxide anion [27]. In addition, TNF-α transcriptionally regulates iNOS and COX2 [28, 29]; swimming-mediated inhibition of TNF-α gene expression may also be a mechanistic link between such molecules and the suppression of colitis.

TNF-α is a pleiotropic cytokine that activates immune cells in IBD and subsequently promotes production of other pro-inflammatory cytokines [30]. IFN-γ also plays a critical role in the induction and progression of colitis [31]. TNF-α- and IFN-γ-expressing CD3^+^ T cells, which are chemo-attracted to and activated by CCL2 and KC, are highly involved in IBD exacerbation [32]. In this study, we showed that DSS-enhanced mucosal CCL2 and KC production, as well as the infiltration of TNF-α- and IFN-γ-expressing CD3^+^ T cells into the MLNs and LPs, was reduced when rats were treated with swimming exercise, suggesting that swimming reduces the levels of inflammatory mediators and mitigates infiltration of the injured site by activated CD3^+^ T cells.

Several clinical and experimental studies [5, 33] have implicated oxidative stress in IBD, in which the surge of ROS and NO generated by activated neutrophils causes intestinal injury. However, appropriate ROS production can be beneficial because specific adaptations are evoked. Such adaptations include increased antioxidant/oxidative damage-repairing enzyme activity and lower levels of oxidative stress [34]. Moreover, NF-κB mediates a major oxidative stress-sensitive signal transduction pathway in mammalian tissues. Appropriate activation of the NF-κB signaling cascade increases expression of genes encoding important enzymes such as mitochondrial SOD, which maintain cellular oxidant/antioxidant homeostasis during exercise [35, 36]. Nevertheless, excessive ROS production would cause overactivation of NF-κB, resulting in disruption of the oxidant/antioxidant balance. In the present study, we demonstrated that swimming prevented intense oxidative stress and boosted antioxidant status in rats with DSS-induced colitis, as evidenced by reductions in ROS, MDA, and NO levels, iNOS activity, and NF-κB activation, in addition to the reinstatement of GSH and TAC levels and SOD and GPx activity. These findings agree with those of previous studies and reinforce the premise that swimming exerts antioxidant effects that protect against tissue damage [37]. It is postulated that swimming reduces the ROS to an appropriate level, and that mild oxidative stress stimulates the expression of certain antioxidant enzymes, which then maintain oxidant/antioxidant homeostasis. However, these assumptions require further study.

Excessive exposure of intestinal mucosa to ROS under inflammatory conditions increases epithelial cell apoptosis and LDH release [15, 38], which likely alters epithelial barrier integrity and contributes to intestinal injury [7]. Apoptosis is partly regulated by the Bcl-2 family, which includes Bcl-2 and Bax. Bcl-2 is regarded as a pro-survival molecule, whereas Bax is a pro-apoptotic molecule as it binds to and antagonizes the effects of Bcl-2 [7]. An increased Bax/Bcl-2 ratio increases the release of AIF and cytochrome c from mitochondria into the cytosol, which then activates caspase-9 and caspase-3, ultimately causing apoptosis [39]. A previous study showed that exercise attenuates age-induced increases in the Bax/Bcl-2 ratio and reduces apoptosis [40]. The data presented herein revealed that swimming attenuated DSS-induced colon cell apoptosis by decreasing caspase-3 activity, reducing AIF, cytochrome c, Bax, and Cl-caspase-3 protein levels, and simultaneously upregulating Bcl-2 expression, thereby ameliorating epithelial barrier dysfunction and improving colon architecture.

The present study has several limitations. First, we did not investigate whether the protective effects of swimming against DSS-induced chronic colitis are frequency- and/or intensity-dependent. Second, we did not undertake a detailed investigation of the potential signaling pathways involved in the process by which swimming protects against DSS-inducted chronic colitis. Other factors, which include aging, animal species, and treatment protocol, also need to be considered if we are planned to fully evaluate the protective effects of swimming.

In summary, we showed that swimming may be an adjunct or alternative therapy for DSS-induced colitis because it exerts anti-inflammatory, antioxidant, and anti-apoptotic effects. The beneficial effects of swimming were associated with its ability to modulate the production of NF-κB, COX-2, and inflammatory mediators. Swimming mitigated oxidative stress and boosted enzymatic/non-enzymatic antioxidant defenses. Moreover, swimming reduced caspase-3 activity and pro-apoptotic gene expression, with concomitant upregulation of Bcl-2. These findings suggest that swimming is an effective therapy for DSS-induced chronic colitis. However, the exact molecular mechanisms and signaling networks underlying the positive effects of swimming still need to be examined. Thus, further studies should investigate the potential therapeutic efficacy of swimming for the management of IBD following the manifestation of symptoms.

## MATERIALS AND METHODS

### Materials

All reagents were obtained from Sigma (St. Louis, MO, USA) unless otherwise noted. All chemicals were of the highest commercial grade available. Rabbit anti-rat COX2, NF-κB p65, p-NF-κB p65 (Ser536), β-actin, and lamin B antibodies were purchased from Abcam (Cambridge, UK). Rabbit anti-rat AIF, Bax, Bcl-2, caspase-3, and Cl-caspase-3 were acquired from Abnova (Taiwan, China). Horseradish peroxidase-conjugated anti-rabbit IgG was procured from Chemicon (Temecula, CA, USA). 2′,7′-Dichlorodihydrofluorescein diacetate, acetyl ester, and dihydroethidium were purchased from Molecular Probes (Eugene, OR, USA). DSS (MW = 36,000 to 50,000; ICN Biomedicals Inc., Aurora, Ohio, USA) was dissolved in regular drinking water. All suspensions were freshly prepared before use.

### Animals and ethics statement

Male Sprague-Dawley rats (6 weeks old and weighing 140 g to 160 g) were obtained from the Laboratory Animal Center of the Fourth Military Medical University and maintained at a comfortable temperature (22°C to 24°C) and relative humidity of 40% to 70%, with a regular 12 h day/night cycle, and access to standard laboratory chow and tap water *ad libitum*. All experimental protocols were approved by the Institutional Animal Research Ethics board of the 150th Central Hospital of PLA. Each animal received humane treatment in full compliance with the National Institutes of Health (Bethesda, MD, USA) criteria for the Care and Use of Laboratory Animals. Rats were sacrificed under anesthesia by intraperitoneal injection of sodium pentobarbital, and all efforts to minimize suffering were made. Euthanasia by sodium pentobarbital was performed in accordance with the American Veterinary Medical Association Guidelines on Euthanasia, June 2007.

### DSS-induced chronic colitis and weight-unloaded swimming exercise

After transport, rats were acclimatized for 7 d. A DSS-induced chronic colitis model was established as previously described [41]. The protocol involved three 5 d cycles of 2% DSS separated by 14 d intervals. Animals without DSS treatment were used as a vehicle control and had access to regular drinking water during the study.

Weight-unloaded swimming training was performed as previously described [37], with slight modifications. Swimming took place in a 120 cm glass container filled with water to a depth of 80 cm and maintained at 35°C to 36°C. Rats were acclimatized to the training pool for 1 week prior to swimming training. Acclimation entailed swimming for gradually increasing times (30, 40, 50, 60, 70, 80, and 90 min/d). After acclimation, rats were trained for 7 weeks (either 1 h/d or 1.5 h/d on 5 d/week). DSS administration began on the first swimming day. Untrained rats were kept in the same glass containers, which contained water (maintained at 35°C) at a depth of 4 cm.

### Sample collection and preparation

After 7 weeks of swimming exercise, rats were fasted for 4 h. Samples of blood was centrifuged for 15 min at 1, 000 × *g*, and serum samples were harvested and stored at -20°C for detection of LDH release and inflammatory mediators. At the time of euthanasia, feces were collected and stool samples were extracted in the extraction buffer (Sigma), centrifuged for 5 min at 13,000 × *g*, and then used for the measurement of calprotectin and lactoferrin. After all animals were euthanized, laparotomy was performed immediately. The spleen was dissected for photoing and weighing. The colon was removed from the distal end of the cecum to the rectum, freed of adherent adipose tissue, split longitudinally, and washed with ice-cold saline to remove fecal residue and photographed. Isolated colons were utilized for histopathological, immunohistochemical, biochemical investigation, and other analyses.

### Experimental design

Ninety rats were randomly divided into six groups (*n* = 15/group) as follows: (1) Vehicle + Sedentary group (Ctrl + Sed) [rats were administered regular drinking water without swimming exercise]; (2) Vehicle + Exercise group 1 (Ctrl + Ex 1) [identical to the Ctrl + Sed group, except that rats swam for 1 h/d]; (3) Vehicle + Exercise group 2 (Ctrl + Ex 2) [identical to the Ctrl + Sed group, except that rats swam for 1.5 h/d]; (4) DSS + Sedentary group (DSS + Sed) [identical to the Ctrl + Sed group, except that rats received 2% DSS/water instead of regular drinking water]; (5) DSS + Exercise group 1 (DSS + Ex 1) [identical to DSS + Sed group, except that rats swam for 1 h/d]; and (6) DSS + Exercise group 2 (DSS + Ex 2) [identical to DSS + Sed group, except that rats swam for 1.5 h/d].

### Measurement of BW, spleen weight and colon length

BW was measured before and after adaptation, and once a week throughout the experimental period. After 7 weeks of swimming exercise, rats were fasted for 4 h and then euthanized with sodium pentobarbital. The spleen and entire colon were removed, and spleen weight and colon length were measured.

### Determination of colon barrier function

Intestinal barrier function was examined as previously described [41]. In brief, colon barrier integrity was assessed according to its permeability to FITC-dextran (MW = 4,000; FD4). After 4 h of fasting, rats received FD4 by gavage [600 mg/kg BW in 0.2 mL of sodium-phosphate buffered saline (PBS)]. Plasma samples were obtained 5 h after the procedure, and fluorescence was measured.

### Histological examination and quantitation of inflammation

Colon tissues were fixed overnight in PBS-buffered 10% formalin, embedded in paraffin, non-serially sectioned (4 µm thick), and mounted on poly-L-lysine-covered slides. After deparaffinization in xylene and rehydration in a graded series of ethanol solutions, the sections were stained with hematoxylin and eosin (HE). Histological changes were evaluated by two independent pathologists as previously described [42]; neither pathologist had any knowledge of the treatment regimen received by each animal. Inflammation was graded according to its extent (focal, multifocal, diffuse, or extensive) and depth/penetration into the LP, submucosa, muscularispropria, and sub-serosa. The degree of inflammation was scored from 0 to 4, where 0 indicates no observable inflammation and 4 denotes severe inflammation and/or ulceration/erosion. Tissue histology was recorded upon observation under a light microscope (Leica, Wetzlar, Germany) equipped with image capturing software. Image-Pro Discovery software (Media Cybernetics Inc., Silver Spring, MD, USA) was used to measure crypt depth, which was determined from 10 to 15 independent measurements on at least three different tissue sections per rat.

### IHC and staining evaluation

After deparaffinization and rehydration, colon sections were submerged in citrate buffer (pH 6.0) and boiled in an autoclave at 121°C for 3 min for antigen retrieval. The slides were then cooled to room temperature. Endogenous peroxidase was quenched with 0.3% H_2_O_2_ in methanol for 15 min. Nonspecific adsorption was minimized by incubating the section in 10% normal goat serum (Gibco, BRL, UK) in PBS for 20 min. Sections were incubated overnight with primary antibodies and then with a biotinylated secondary antibody (ChemMateEnVision Kit; DAKO, Hamburg, Germany) for 15 min. The reaction products were visualized using diaminobenzidine (DAB) substrate (Maixin Biotech., Fuzhou, China). Sections were counterstained with commercial hematoxylin (Maixin Biotech.), dehydrated, and examined under a light microscope (Leica).

The NF-κB p65 and COX2 protein levels were evaluated by a blind way in 10 fields under 100× magnification from each slide. IHC staining was scored as previously described [43]. One hundred cells per field were categorized as follows: “0”, 0%, no staining; 1 to 25% of cells were stained; “2”, 26 to 50% of cells were stained; and “3”, > 50% of cells were stained.

### Measurement of MPO activity

MPO activity, an indicator of neutrophil infiltration into the colon tissue, was measured using commercial kits from Molecular Probes (Eugene, OR, USA), as previously described [44]. Briefly, approximately 1 g of wet colon tissue was homogenized in 0.5% hexadecyltrimethylammonium bromide dissolved in PBS. The tissue homogenate was centrifuged at 12,500 × *g* for 40 min at 4°C, and the supernatant was collected to detect MPO activity, which was defined by a change in the absorbance measured by a spectrophotometer (DU 640B; Beckman) at 590 nm. Results were expressed as absorbance units per gram of wet tissue.

### Flow cytometry analysis

Cells were freshly isolated from MLN and LP, as previously described [16]. Fc receptors on the cells were pre-blocked at 4°C for 15 min. The cells were washed with fluorescence-activated cell sorting (FACS) staining buffer (PBS containing 1% bovine serum albumin) and stained with Cy-conjugated anti-CD3 (145-2C11) and FITC-conjugated Ly6G (neutrophils; BD Pharmingen) antibodies at 4°C for 30 min, with occasional shaking. The cells were washed twice with FACS staining buffer, resuspended in BD Cytofix/Cytoperm (BD Pharmingen) solution for 20 min, and then washed twice in BD Perm/Wash solution. To examine intracellular cytokine expression, the resuspended, fixed, and permeabilized cells were stained with predetermined allophycocyanin fluorochrome-conjugated anti-cytokine antibodies (anti-TNF-α and anti-IFN-γ) at 4°C for 30 min in the dark. Lymphocytes were thoroughly washed with FACS staining buffer and analyzed by flow cytometry (BD Aria П, Becton Dickinson, USA).

### Measurement of serum LDH

The blood samples collected from all animals were centrifuged, serum separated and used to determine LDH activity using BioVision kit (Milpitas, CA, USA).

### ELISAs

The entire colon was removed from the distal end of the cecum to the rectum, cut longitudinally, thoroughly rinsed in sterile 20 mM PBS (pH 7.4), spread onto the bottoms of six-well dishes, and incubated overnight in 1 mL of culture medium. The supernatant levels of TNF-α, IL-1β, IL-6, IL-10, KC, CCL2, and PGE_2_ and serum levels of TNF-α, IL-1β, IL-6, KC, and PGE_2_ were measured using commercial ELISA kits (R&D Systems, Minneapolis, MN, USA). Fecal levels of calprotectin and lactoferrin were determined using ELISA kits (Abbexa, Cambridge, UK). Optical density (OD) was measured on an ELISA plate scanner (CA94089, Molecular Devices, Sunnyvale, Canada).

### Measurement of ROS production

ROS levels were determined by measuring the oxidative conversion of DCFH-DA to the fluorescent compound DCF. Briefly, colon tissues were homogenized in PBS (pH 7.4) and centrifuged at 10,000 × *g* for 20 min at 4°C, and the supernatant was collected, washed with warm HBSS, and incubated in HBSS containing 10 μM DCFH-DA at 37°C for 30 min. DCF fluorescence was determined at 485 nm (excitation) and 520 nm (emission) in a fluorescence microplate reader (Safire2, Tecan, Switzerland).

### Measurement of MDA

MDA levels were determined using the thiobarbituric acid assay as reported by Buege and Aust [45]. Absorbance was recorded at 535 nm, and the results were expressed as nmol/g tissue.

### Measurement of iNOS activity

iNOS activity was measured by monitoring the conversion of arginine to citrullinein, as previously described [46]. Briefly, an aliquot of colon tissue lysate was incubated with L- [^3^H] arginine along with essential substrates and cofactors (tetrahydrobiopterin, nicotinamide adenine dinucleotide phosphate, and flavin adenine dinucleotide). The amount of product, namely, L- [^3^H] citrulline, was calculated after liquid scintillation counting. To quantify iNOS activity, ethylene diamine tetraacetic acid (EDTA) and ethylene glycol tetraacetic acid (EGTA) were added sequentially to the incubation buffer. An appropriate blank, including 1 mM L-NAME, a competitive iNOS inhibitor, was used to exclude the effects of nonspecific L-arginine metabolism. The iNOS activity in the citrulline assay was analyzed according to the degree of L-NAME inhibition in the EDTA-EGTA sample, and its expression was measured in Units (1 Unit = 1 pM L-citrulline/mg protein/min).

### Measurement of nitrite

The production of nitrite, an indicator of NO synthesis, was measured in a colorimetric reaction with the Griess reagent [47]. Briefly, colon tissues were homogenized in PBS (pH 7.4) and centrifuged at 10,000 × *g* at 4°C for 20 min. The supernatant was collected and mixed with equal (1:1) volumes of Griess reagent [0.1% N-(1-naphthyl) ethylenediaminedihydrochloride, 1% sulfanilamide, and 2.5% H_3_PO_4_]. A 96-well microplate reader (Spectra MAX 340PC; Molecular Devices) was used to measure the absorbance at 540 nm. Data were analyzed using Softmax Pro software. Sodium nitrite, dissolved in double-distilled water, was used as a standard.

### Measurement of GSH

GSH levels in the supernatants of colon homogenates were determined using 5,5′-dithiobis 2-nitrobenzoic acid (DTNB) reagent as previously described [48]. The OD of the colored product was read at 412 nm, and the results were expressed as nmol/g tissue.

### Determination of TAC

TAC was determined using the Cayman total antioxidant assay kit, according to the manufacturer's instructions. The assay depends on the ability of antioxidants in the supernatants of colon homogenates to inhibit the metmyoglobin-mediated oxidation of ABTS [2,2-azino-di-(3-ethylbenzthiazoline sulfonate)]. The amount of oxidized product was estimated by measuring the absorbance at 405 nm. The antioxidant capacity of the sample was compared with that of Trolox, a water-soluble tocopherol analogue, and the results were expressed as mmol of Trolox equivalent/g tissue.

### Measurement of SOD activity

SOD activity was determined by the ability of colonic SOD to inhibit the auto-oxidation of pyrogallol, as previously described [49]. The change in absorbance at 420 nm was measured at 1 min intervals for 3 min. One unit of SOD is defined as the amount of enzyme that affords 50% inhibition of pyrogallol auto-oxidation in 1 min. The results were expressed as U/mg protein.

### Determination of GPx activity

GPx activity was determined according to the method of Paglia and Valentine [50]. This method depends on the ability of the enzyme to oxidize GSH, which was monitored by recording the decrease in absorbance of NADPH at 340 nm. One unit of enzyme is defined as the amount of enzyme that oxidizes 1 mmol NADPH/min at 25°C.

### Caspase-3 activity assay

Caspase-3 activity was detected using the Caspase-3/CPP32 Colorimetric Assay Kit (BioVision), according to the manufacturer's instructions. An aliquot of homogenate supernatant was incubated with the labeled substrate, DEVD-pNA (acetyl-Asp-Glu-Val-Asp p-nitroanilide). Cleavage of the peptide by the caspase releases the chromophore, pNA. Results were read at 405 nm in a microplate reader (Bio-Tek instruments Inc., Winooski, VT, USA) and expressed as the fold change in caspase-3 activity.

### Western blot analysis

Colon tissue samples were homogenized in tissue lysis and extraction buffer containing protease inhibitor cocktail set III (EMD Chemicals Inc., Germany). Crude homogenates were repetitively filtered and centrifuged at 15,000 × *g* at 4°C for 25 min. The supernatants were collected as cytosolic fractions. The nuclei-rich pellets were resuspended in nuclear protein extraction buffer containing 20 mM 4-(2-hydroxyethyl)-1-piperazineethanesulfonic acid (pH 7.9), 10% glycerol, 1 mM dithiothreitol, 400 mM NaCl, 1 mM EDTA, and cocktail set III. After 20 min of centrifugation at 15,000 × *g* at 4°C, the supernatants containing nuclear proteins were pooled. NF-κB p65 levels in the nuclear fractions and the levels of all other proteins in the cytosolic fractions were quantified. The two extracts (cytosolic and nuclear) were boiled, and the proteins were separated by sodium dodecyl sulfate polyacrylamide gel electrophoresis, electrotransferred onto nitrocellulose membranes, and immunoblotted with appropriate primary antibodies. Equivalent sample loading was confirmed by probing with rabbit monoclonal antibodies against rat β-actin and lamin B. Bands were detected using an enhanced Chemiluminescence Assay Kit (Pierce, Rockford, IL, USA).

### Statistical analysis

All data were expressed as the mean ± standard derivation (SD). Intergroup differences were analyzed using Student's *t*-test (two-tailed, unpaired) or one-way ANOVA, followed by Dunnett's post-hoc test where appropriate. GraphPad statistical software (GraphPad Software, Inc., San Diego, CA, USA) was used for data analysis. *P* < 0.05 was considered to be statistically significant.

## Abbreviations

AIF: apoptosis-inducing factor
BW: body weight
Cl-caspase-3: cleaved-caspase-3
COX2: cyclooxygenase 2
DAB: diaminobenzidine
DSS: dextran sulfate sodium
ELISA: enzyme-linked immunosorbent assay
FACS: fluorescence-activated cell sorting
FD4: FITC-dextran
GPx: glutathione peroxidase
GSH: glutathione
HE: hematoxylin and eosin
IBD: inflammatory bowel disease
IFN-γ: interferon-γ
IHC: Immunohistochemistry
IL: interleukin
iNOS: inducible nitric oxide synthase
LP: lamina propria
MDA: malondialdehyde
MLN: mesenteric lymph nodes
MPO: myeloperoxidase
NF-κB: nuclear factor-κB
NO: nitric oxide
PBS: sodium-phosphate buffer
PGE_2_: prostaglandin E_2_
ROS: reactive oxygen species
SOD: superoxide dismutase
TAC: total antioxidant capacity
TNF-α: tumor necrosis factor-α
UC: ulcerative colitis
